# Strong interaction between T allele of endothelial nitric oxide synthase with B1 allele of cholesteryl ester transfer protein TaqIB highly elevates the risk of coronary artery disease and type 2 diabetes mellitus

**DOI:** 10.1186/1479-7364-6-20

**Published:** 2012-09-25

**Authors:** Zohreh Rahimi, Reza Nourozi-Rad, Ziba Rahimi, Abbas Parsian

**Affiliations:** 1 Medical Biology Research Center, Medical School, Kermanshah University of Medical Sciences, Daneshgah Avenue, P.O. Box 67148–69914, Kermanshah, Iran; 2 Department of Biochemistry, Medical School, Kermanshah University of Medical Sciences, Daneshgah Avenue, P.O. Box 67148–69914, Kermanshah, Iran; 3 Department of Biochemistry, Medical School, Dezful University of Medical Sciences, Azadegan Avenue, P.O. Box 6461653476, Dezful, Iran; 4 Division of Neuroscience & Behavior, NIAAA, National Institutes of Health, Rockville, Maryland, 20852, USA

**Keywords:** NOS3 G894T, CETP TaqIB, CAD, T2DM, Western Iran

## Abstract

**Background:**

The present study was conducted to investigate the possible outcome of interaction between endothelial nitric oxide (NOS3) G894T and cholesteryl ester transfer TaqIB variants on the risk of coronary artery disease (CAD) and type 2 diabetes mellitus (T2DM). The sample included a total of 207 CAD patients (102 CAD patients with T2DM and 105 CAD patients without T2DM). There were also 101 patients with T2DM and 92 age- and sex-matched healthy individuals as controls. All study participants were from Western Iran. The sample was genotyped by polymerase chain reaction-restriction fragment length polymorphism.

**Results:**

The presence of NOS3 T allele was not associated with the risk of CAD or T2DM, and the CETP B1 allele was only significantly associated with the increased risk of CAD in total CAD patients (odds ratio (OR) = 5.1, *p* = 0.019). However, the concomitant presence of both CETP B1 and NOS3 T alleles significantly increased the risk of CAD in total CAD patients (OR = 18.1, *p* < 0.001), in CAD patients without T2DM (OR = 27.1, *p* = 0.03), and in CAD patients with T2DM (OR = 13.5, *p* = 0.002). Also, the presence of both alleles increased the risk of T2DM (OR = 12, *p* = 0.004).

**Conclusions:**

Our findings, for the first time, indicate that NOS3 T allele strongly interacts with CETP B1 allele to augment the risk of CAD and T2DM in the population of Western Iran.

## Introduction

Cholesteryl ester transfer protein (CETP) participates in reverse cholesterol transport by transfering cholesteryl esters from high-density lipoprotein-cholesterol (HDL-C) to apolipoprotein-B containing particles in exchange for triglycerides (TG), thereby reducing the concentration of HDL-C [1-3].

CETP gene located on chromosome 16q21 consists of 16 exons and 15 introns and has several genetic polymorphisms affecting CETP activity among which TaqIB polymorphism has been most widely studied. This polymorphism results from G to A base pair change at nucleotide 277 in intron 1 of the CETP gene which disrupts TaqI restriction site (single-nucleotide polymorphism (SNP) rs708272). The allele containing the TaqI endonuclease site is called B1, while the allele without the restriction site is called B2. The less common B2 allele has been associated with increased HDL-C levels and decreased CETP activity and levels [4,5]. The association of CETP variants with coronary artery disease (CAD) and type 2 diabetes mellitus (T2DM) has been investigated in several studies without consistent results [1,6-9].

Nitric oxide synthase (NOS) synthesizes NO through the oxidation of l-arginine to l-citrulline which is an important regulator of vasodilator tone and blood pressure [10,11]. One of the most clinically important polymorphisms of NOS3, the G894T polymorphism, results from G to T conversion at nucleotide 894 of exon 7 of gene that changes Glu298Asp (SNP rs1799983). The presence of this polymorphism reduces the NO production. There are several studies reporting controversial results related to the role of NOS3 variants on the susceptibility to CAD and T2DM [12-17].

Recently, we reported that the B1 allele of CETP increased the risk of CAD and T2DM 1.65- and 1.7-fold, respectively [18]. Also, we indicated that the risk of CAD was increased by 2.15-fold in the presence of T allele of NOS3 in our population [19]. To our knowledge, there is no report related to the influence of concomitant presence of CETP variants and NOS3 G894T polymorphism on the risk of CAD and T2DM in the literature.

The aim of the present study was to examine the interaction between CETP TaqIB and NOS3 G894T variants and its effect on the risk of CAD and T2DM in the population of Western Iran.

## Materials and methods

The sample consisted of 308 patients and 92 controls. There were 207 total CAD patients including 113 males and 94 females with the mean age of 56.9 ± 8.6 years from whom there were 102 CAD patients with T2DM (CAD/T2DM) and 105 CAD patients without T2DM. There were 101 patients with T2DM including 51 males and 50 females with the mean age of 56.5 ± 9.8 years. The controls were 92 non-diabetic individuals that consisted of 47 males and 45 females with the mean age of 54.3 ± 8.5 years who were evaluated by angiography for suspected CAD but had normal coronary arteries. All patients and controls were recruited from Imam Ali Hospital of Kermanshah University of Medical Sciences and were from Western Iran with Kurdish ethnic background.

Patients were referred to the Cardiology Division of the Imam Ali Hospital of the Kermanshah University of Medical Sciences to undergo their first coronary angiography to evaluate the presence and extent of CAD. Coronary artery involvement was defined as a ≥50% diameter obstruction of a major coronary vessel. The diagnosis of diabetes in patients was confirmed using WHO criteria [20]. Written informed consent was obtained from each individual before participation in the study. The study was approved by the Ethics Committee of Kermanshah University of Medical Sciences and was in accordance with the principles of the Declaration of Helsinki (2000).

### Genotype analysis

DNA was extracted from the leukocytes of the whole blood by the phenol-chloroform method as previously described in [21]. The NOS3 G894T polymorphism was detected by amplification of a region in exon 7 of the gene using the forward primer of 5^′^-AAG GCA GGA GAC AGT GGA TGG A-3^′^ and the reverse primer of 5^′^-CCC AGT CAA TCC CTT TGG TGC TCA-3^′^ and subsequent digestion with MboI restriction enzyme as previously described in [22].

For the detection of the CETP TaqIB genotypes, the following primers were used: 5^′^-CACTAGCCCAGAGAGAGGAGTGCC-3^′^ and 5^′^ CTGAGCCCAGCCGCACACTAAC-3^′^ to amplify the 535-bp fragment in intron 1 of the CETP gene which was amplified by PCR, and the products were digested with TaqIB restriction endonuclease as previously described in [18].

### Chemical analysis

Plasma total cholesterol (TC) and TG were measured by the standard enzymatic method (Pars Azmoon kit, Pars Azmoon Inc., Tehran, Iran), using an automated Technicon RA-1000 (Technicon Instruments Corporation, NY, USA). The plasma low-density lipoprotein-cholesterol (LDL-C) and HDL-C levels were measured using commercially available enzyme assay kits (Pars Azmoon kit, Iran).

### Statistical analysis

The allelic frequencies were calculated by the chromosome counting method. The genotype and allele frequencies of CETP and NOS3 in CAD and T2DM patients were compared to controls, and the significance of differences calculated using *χ*^2^ test. Odds ratios (OR) were calculated as estimates of relative risk for disease and 95% confidence intervals (CI) obtained by SPSS logistic regression software. A two-tailed Student's *t* test was used to compare quantitative data. Statistical significance was assumed at the *p* < 0.05 level. The SPSS statistical software package version 16 was used for the statistical analysis.

## Results

The biochemical characteristics of patients and controls are presented in Table 1. The plasma level of LDL-C and TG was significantly higher, and HDL-C level was significantly lower in both CAD and T2DM patients compared to controls. In Table 2, the lipid profile of CAD/T2DM patients has been compared to CAD, T2DM, and controls. Significantly higher level of TG and lower level of HDL-C were observed in CAD/T2DM patients compared to those in CAD patients and controls. Although the level of LDL-C was higher in CAD/T2DM patients compared to those in CAD and control groups, the differences reached statistically significant values only with controls. Plasma total cholesterol was insignificantly increased in CAD/T2DM group compared to those in all studied groups. The same results were obtained when the levels of TG, HDL-C, and LDL-C were compared between T2DM patients with CAD and controls. In CAD patients, the level of LDL-C was significantly increased when compared to that in controls (Table 2). The plasma lipid and lipoprotein profiles categorized based on gender in each group are shown in Table 2. Comparison of genders in each group for lipid and lipoprotein levels indicated that women with T2DM had significantly higher plasma total cholesterol than males with T2DM (Table 2). Table 3 indicates the synergistic effect of both CETP B1 and NOS3 T alleles on the risk of CAD and T2DM. The concomitant presence of both alleles increased the risk of CAD significantly by 27.1-, 18.1-, and 13.5-fold in CAD patients without T2DM, total CAD, and CAD patients with T2DM, respectively. Also, the presence of both alleles (B1 and T) increased the risk of T2DM by 12-fold (*p* = 0.004) (Table 3). However, as demonstrated in Table 3, the presence of NOS3 T allele alone did not increase the risk of CAD or T2DM. Further, the presence of CETP B1 allele was significantly (*p* = 0.019) associated with 5.1-fold increased risk of CAD in total CAD patients.

**Table 1:** Demographic and biochemical parameters of the controls and patients

**Parameters**	**CAD patients with and without diabetes (*n*** **= 207)**	**T2DM (*n*** **= 101)**	**Control subjects (*n*** **= 92)**
Age (years)	56.9 ± 8.6*NS	56.5 ± 9.8	54.3 ± 8.5
Sex (M/F)	113/94*NS	51/50	47/45
FBS (mg/dl)	136.2 ± 59.6, *p* < 0.001	162.9 ± 71.3, *p* < 0.001	97.0 ± 20.1
History of hypertension (%)	102 (49.6), *p* < 0.001	29 (28.7), *p* = 0.68	24 (26.1)
LDL-C (mg/dl)	96 ± 28, *p* < 0.001	98.5 ± 38.4, *p* < 0.001	83 ± 18
HDL-C(mg/dl)	47 ± 9.3, *p* = 0.007	45.2 ± 10.2, *p* < 0.001	49.8 ± 5.8
TC (mg/dl)	186 ± 42, *p* = 0.046	185.9 ± 45.8, *p* = 0.09	177 ± 26
TG (mg/dl)	177 ± 89, *p* = 0.038	200.8 ± 99.3, *p* = 0.001	156 ± 48
BMI (kg/m^2^)	26 ± 2.7*NS	26.2 ± 2.7*NS	26.6 ± 3.7

Plasma fasting blood sugar (FBS), LDL-C, HDL-C, TC, and TG levels, as well as age and BMI were compared between patients and controls using two-tailed Student’s *t* test. The sex was compared between two groups by the *χ*^2^-test. NS, not significant; *p > 0.05.

**Table 2:** Comparison of plasma lipid and lipoprotein levels in patients and controls

**Patients (*n*)**	**TG (mg/dl)**	**TC (mg/dl)**	**LDL-C (mg/dl)**	**HDL-C (mg/dl)**
CAD with T2DM	202.2 ± 87.9*	207.6 ± 185.4	97.8 ± 30.8**	44.7 ± 10.5*
Males (52)	206.4 ± 85.9	225.8 ± 253.6	98.6 ± 25.9	43.8 ± 8.2
Females (50)	197.8 ± 90.6	188.6 ± 56.2	96.8 ± 35.3	45.6 ± 12.5
CAD without T2DM	151.8 ± 83.2***	182.8 ± 36.1	94.1 ± 25.9**	49.2 ± 7.5***
Males (61)	150.3 ± 93.3	184.2 ± 37.7	97.2 ± 27.6	48.2 ± 8.2
Females (44)	153.9 ± 67.5	180.8 ± 34.1	89.8 ± 23.1	50.4 ± 6.1
T2DM	200.8 ± 99.3*	185.9 ± 45.8	98.5 ± 38.4**	45.2 ± 10.2*
Males (51)	198.4 ± 110.6	175.9 ± 41.2****	90.9 ± 31.3*	42.5 ± 9.7*
Females (50)	203.2 ± 87.1	196.2 ± 48.3	106.2 ± 43.3	47.9 ± 10.1
Controls	161.5 ± 64.5	176.8 ± 25.8	82.8 ± 17.8	49.8 ± 5.8
Males (47)	161.1 ± 53.5	179.7 ± 27.2	85.3 ± 17.4	48.4 ± 5*
Females (45)	161.8 ± 74.8	173.7 ± 24.2	80 ± 17.9	51.3 ± 6.1

*p < 0.05 compared to CAD without T2DM and controls; **p < 0.05 compared to controls; ***p < 0.05 compared to CAD with T2DM and T2DM; ****p < 0.05 compared between males and females in each group.

**Table 3:** The carrier odds ratio interaction between CETP B1 and NOS3 894 T alleles

**CETP B1**	**NOS3 T**	**Total CAD patients *n *(%) ORs (95% CI) *n *** **= 207**	**CAD with T2DM *n *(%) ORs (95% CI) *n *** **= 102**	**CAD without T2DM *n *(%) ORs (95% CI) *n *** **= 105**	**T2DM patients *n*(%) ORs (95% CI) *n *** **= 101**	**Controls *n *(%) *n *** **= 92**
−	−	3 (1.4)	2 (2)	1 (1)	2 (2)	8 (8.7)
−	+	3 (1.4)	3 (2.9)	0	2 (2)	12 (13)
		0.67 (0.11–4.17, *p* = 0.67)	1 (0.14–7.4, *p* = 1)		0.67 (0.1–5.7, *p* = 0.71)	
+	−	113 (54.6)	53 (52)	60 (57.1)	58 (57.4)	59 (64.2)
		5.1 (1.3–20, *p* = 0.019)	3.6 (0.73–17.7, *p* = 0.11)	8.14 (1–67, *p* = 0.05)	3.9 (0.8–19.3, *p* = 0.09)	
+	+	88 (42.6)	44 (43.1)	44 (41.9)	39 (38.6)	13 (14.1)
		18.1 (4.2–76.8, *p* < 0.001)	13.5 (2.6–71.8, *p* = 0.002)	27.1 (3.1–237, *p* = 0.003)	12 (2.25–63.8, *p* = 0.004)	

Interaction with respect to B2 or G alleles in CAD and T2DM patients compared with controls. Overall distribution of interaction between CETP B1 and NOS3 T alleles in total CAD compared to controls (*χ*^2^ = 42.4, df = 3, *p* < 0.001) and between CAD with T2DM compared to controls (*χ*^2^ = 25.7, df = 3, *p* < 0.001), as well as comparison between CAD without T2DM and controls (*χ*^2^ = 33.6, df = 3, *p* < 0.001). Overall distribution of interaction between CETP B1 and NOS3 T alleles in T2DM patients compared to controls (*χ*^2^ = 23.4, df = 3, *p* < 0.001).

## Discussion

Diabetes mellitus is a strong risk factor for the development of atherosclerosis [6] that is related to dyslipidemia linked to insulin resistance characterized by a high triglyceride level, a high LDL-C, and a low HDL-C level [23]. In fact, decreased concentrations of HDL-C have been reported to be significantly related to CAD in patients with type 2 diabetes mellitus [7]. The significant higher level of TG and lower level of HDL-C observed in CAD patients with T2DM compared to those in CAD patients without diabetes in the present study confirm the role of dyslipidemia for predicting the risk of CAD in T2DM patients.

Both genetics and environmental factors have important roles in the pathogenesis of CAD and T2DM [24]. The role of genetic factors appears to be considerably different between various populations [25].

The most commonly studied polymorphism of CETP is TaqIB with a frequency of 0.44 in Caucasian populations and is associated with decreased CETP mass, an increase in HDL-cholesterol, and modulating the risk for diabetic complications in patients with T2DM. The presence of homozygous B1 allele is associated with lowest while in B2B2 carriers there is highest HDL-cholesterol concentration [5,26]. The role of CETP TaqIB polymorphism on the risk of CAD and T2DM either through its influence on lipid metabolism or independent of its effect on lipid profile has been reported in various populations [1,6-9]. Previously, we reported an association between the B1 allele of CETP and the risk of CAD and T2DM independent of HDL-C level [18]. Endothelial NOS gene through coding endothelial NOS enzyme has a role in maintaining normal endothelial function. Endothelial NOS through synthesis of NO affects the relaxation of vascular smooth muscle, inhibition of adhesion of platelets and leukocytes to the endothelium, reduction of vascular smooth muscle cell migration and proliferation, and limitation of the oxidation of LDL-C [27,28]. There are several reports indicating an association between the NOS3 G894T polymorphism and the risk of CAD [13,14]. However, some studies demonstrated the lack of association between this polymorphism and the risk of CAD [15,17,29]. Our recent work indicated that the risk of CAD is elevated in the presence of NOS3 T allele [19]. In the present work, we examined the influence of concomitant presence of NOS3 T and CETP B1 alleles on the risk of CAD and T2DM. We noticed that the risk of CAD augments in the presence of both alleles in total CAD and in CAD patients with and without T2DM to the level of 18.1-, 13.5-, and 27.1-fold, respectively. Also, the combined presence of both alleles increased the risk of T2DM by 12-fold. Our study demonstrated that the presence of variations in more than one gene play an important role in the susceptibility to CAD and T2DM.

The presence of hypercholesterolemia and oxidized LDL-C downregulates NOS expression and rapid degradation of NO. The exact mechanism of influence of CETP variants and its interaction with NOS3 G894T polymorphism on the risk of CAD and T2DM is unclear. However, it has been suggested that contribution of CETP to the formation of small dense LDL-C particles and impairment of NO synthesis in the presence of NOS3 G894T variants that are enhanced in hypercholesterolemia might lead to endothelial dysfunction and atherosclerosis [30].

Briefly, our study indicated that the B1 allele of CETP TaqIB polymorphism in combination with NOS3 T allele augments the risk of CAD and T2DM in our population. The results of the present study emphasize the role of gene-gene interaction in the pathogenesis of complex disorders such as CAD and T2DM that need to be confirmed in further studies from other populations.

## Competing interests

The authors declare that they have no competing interests.

## Authors’ contributions

ZoR designed and supervised the research, analyzed and interpreted the data, and drafted the manuscript. RNR and ZiR carried out the molecular genetic studies. AP revised the manuscript. All authors read and approved the final manuscript.
